# FVB/NJ strain as a mouse model for cutaneous leishmaniasis by *Leishmania (L.) amazonensis*

**DOI:** 10.1590/0074-02760230182

**Published:** 2024-03-15

**Authors:** Guilherme Moreira Paiva Carrara, Beatriz Simonsen Stolf

**Affiliations:** 1 Universidade de São Paulo, Instituto de Ciências Biomédicas, Departamento de Parasitologia, São Paulo, SP, Brasil

**Keywords:** FVB/NJ mouse strain, infection, Leishmania (L.) amazonensis, tegumentary leishmaniasis

## Abstract

**BACKGROUND:**

Leishmaniases encompass a spectrum of neglected diseases caused by parasites of the genus *Leishmania*, grouped in two forms: tegumentary and visceral leishmaniasis.

**OBJECTIVES:**

In this study, we propose Friend Virus B NIH Jackson (FVB/NJ) mouse strain as a new experimental model of infection with *Leishmania (Leishmania) amazonensis*, the second most prevalent agent of tegumentary leishmaniasis in Brazil.

**METHODS AND FINDINGS:**

We performed *in vitro* infections of FVB/NJ macrophages and compared them with BALB/c macrophages, showing that BALB/c cells have higher infection percentages and a higher number of amastigotes/cell. Phagocytosis assays indicated that BALB/c and FVB/NJ macrophages have similar capacity to uptake parasites after 5 min incubations. We also investigated promastigotes’ resistance to sera from FVB/NJ and BALB/c and observed no difference between the two sera, even though FVB/NJ has a deficiency in complement components. Finally, we subcutaneously infected FVB/NJ and BALB/c mice with 2 × 10^6^ parasites expressing luciferase. Analysis of lesion development for 12 weeks showed that FVB/NJ and BALB/c mice have similar lesion profiles and parasite burdens.

**MAIN CONCLUSIONS:**

This work characterises for the first time the FVB/NJ mouse as a new model for tegumentary leishmaniasis caused by *Leishmania (L.) amazonensis*.

Leishmaniases constitute a spectrum of diseases caused by protozoan parasites of the genus *Leishmania*, transmitted by the bite of infected female phlebotomine sandflies.^1^ According to the World Health Organization (WHO), leishmaniases are endemic in 99 countries and territories, with more than 1 billion people at risk of infection.^2^ The estimated number of annual cases of the diseases varies from 700,000 to 1 million.^2^

The main clinical forms of leishmaniases are visceral leishmaniasis (VL) and tegumentary leishmaniasis (TL). TL is the most frequent form, and is further subdivided into localised cutaneous, mucosal/mucocutaneous, diffuse cutaneous and disseminated cutaneous.^3^

Infection in humans is caused by more than 20 species of *Leishmania*, most of which belong to subgenera *Leishmania* and *Viannia* and are transmitted by over 90 species of phlebotomine sandflies.^2^ ^,^ ^4^ The most common vectors of *Leishmania* species involved in human disease are species of *Phlebotomus* in the Old World and *Lutzomyia* in the New World.^5^

*Leishmania* species have a digenetic life cycle, with an extracellular phase in the vector’s intestine and an intracellular phase in the vertebrate host.^6^ Transmission to mammals occurs when the female phlebotomine regurgitates metacyclic promastigotes into the skin during blood feeding.^7^ ^,^ ^8^ The promastigotes are phagocytosed by macrophages and other mononuclear phagocytic cells and differentiate into amastigotes, which survive and multiply through simple division within a phagolysosome. Macrophages usually rupture, releasing amastigotes that are phagocytosed by other cells.^3^ ^,^ ^9^ Contamination of the vector occurs when female sandflies ingest infected phagocytes during blood feeding. In the vector’s intestine, amastigotes differentiate into procyclic promastigotes, which undergo metacyclogenesis generating metacyclic promastigotes, which migrate to the proboscis, being regurgitated during a new blood meal.^8^ ^,^ ^10^ ^,^ ^11^

Depending on the host and on the *Leishmania* species, the parasite multiplies in epithelial macrophages or in macrophages in secondary lymphoid organs, potentially causing TL or VL, respectively.^10^ ^,^ ^12^

Over the past 50 years, various murine strains have been employed as models for TL and VL. These models have been used for studies about cell types, cytokines, anti-*Leishmania* effector mechanisms and drugs, as well as for evaluating clinical disease resolution, resistance to secondary infection, and vaccine development.^13^ ^,^ ^14^ The pattern and severity of infection in mice depend on the *Leishmania* species and on the mouse strain. These models reproduce many aspects of human diseases, with a spectrum of susceptibility depending on the mouse strain.^15^ Mouse models are most used for studying TL pathogenesis owing to the high availability of cellular markers and inbred, congenic, and transgenic strains.^16^ Mice of different genotypes inherently display diverse susceptibility to a wide variety of *Leishmania* strains and species, as observed in non-healing and self-healing models.^17^ Infection with *L. (L.) major* stands out as the most extensively investigated experimental model for TL,^13^ in which polarised Th2 x Th1 responses are observed in BALB/c and C57BL/6 strains, respectively.^18^ Other species, such as *L. (L.) amazonensis*, induce different responses according to the mouse strain, but do not display Th1 x Th2 polarization.^19^ ^,^ ^20^

The Friend Virus B NIH Jackson (FVB/NJ) mouse strain, derived from Swiss N:GP (NIH, 1935), is known for its sensitivity to the B strain of Friend murine leukaemia virus.^21^ This strain is commonly used for the development of transgenic lineages due to its docile behaviour, well-defined inbred background, large litters and the development of numerous pronuclei, which facilitate microinjection of DNA.^21^ These mice exhibit deficiency in the C5 factor of the complement system.^22^ Indeed, the Swiss strain (SWR/J) has a deletion of two base pairs near the 5’ terminal region of the C5 gene, resulting in a truncated translation of the C5 protein, which is not functional.^22^ The membrane attack complex (MAC) is an effector of the complement system that generates pores on the pathogen’s surface, leading to their death. MAC assembly initiates by the cleavage of C5-by-C5 convertase, generating C5a and C5b. C5a is an anaphylatoxin with pro-inflammatory and chemotactic effects, while C5b initiates MAC assembly on the pathogen’s surface. A deficiency in C5 thus results in failure in MAC assembly.^23^ On the other hand, opsonisation of *Leishmania* by C3bi, generated after cleavage of C3b, is important for parasite binding to macrophage CR3, and consequently for phagocytosis and survival.^24^ ^,^ ^25^ Indeed, parasites incubated with C5-deficient mouse serum were more efficiently internalised than non-opsonised parasites.^25^ Parasite factors such as ISP2 (inhibitor of serine peptidase 2) were shown to inhibit the formation of C3 and C5 convertase, thus reducing the formation of MAC and the lysis of *L. (L.) donovani* promastigotes.^26^ It was also previously reported that C5a is crucial for the recruitment of neutrophils to draining lymph nodes following *L. mexicana* infection.^27^ These data show the importance of the complement system in *Leishmania* infection.

Regarding the regulatory immune cells, it was observed that the phenotype and function of regulatory T cells (Tregs) varies among BALB/c, C57BL/6 and FVB/NJ.^28^ Indeed, FVB/NJ splenic Tregs secrete IL-10 at higher levels than C57BL/6 Treg cells. Besides, FVB/NJ Tregs perform an optimal suppression in a cell-cell contact, while C57BL/6 cells do not. FVB/NJ CD4+ splenocytes have lower expression of the master Treg transcription factor Foxp3 than C57BL/6 and BALB/c, which have similar levels.^28^

FVB/NJ mice have been employed in studies involving cancer, neurodegenerative diseases, metabolic disorders, autoimmune conditions, and infectious diseases. Among the microorganisms and parasites analysed in this mouse strain we can cite *Helicobacter pylori*,^29^ Influenza,^30^ *Echinostoma Hortense*,^31^ *Heligmosomoides polygyrus*,^32^ *Staphylococcus aureus*,^33^ *Nippostrongylus brasiliensis*,^34^ *Litomosoides sigmodontis*,^35^ *Trypanosoma cruzi*,^36^ *Clonorchis sinensis*,^37^ ^,^ ^38^ *Mycobacterium ulcerans*,^39^ *Pneumocystis*,^40^ and Zika virus.^41^ These studies indicate that FVB/NJ mice exhibit varying susceptibility depending on the pathogen used, generally triggering a strong inflammatory response. In infections with *T. cruzi*, diffuse inflammatory infiltration of mononuclear cells was observed in cardiac tissues, with development of acute myocarditis.^36^ In viral infections with Influenza and Zika, FVB/NJ mice showed reduced body temperature, locomotor activity, and neuroinflammatory changes in foetuses.^30^ ^,^ ^41^ Upon bacterial infections, FVB/NJ mice were permissive to *S. aureus* after neutrophil depletion.^33^ *H. pylori* and *M. ulcerans* triggered a strong innate immune response, without clinical signs and with spontaneous healing of lesions after the ulcerative phase, respectively.^29^ ^,^ ^39^ For *M. ulcerans*, there was reduced inflammation and infiltration of myeloid cells in draining lymph nodes, without involvement of humoral response in pathogen clearance and protection.^39^ Considering worm infections, FVB/NJ showed resistance to *E. hortense*, *N. brasiliensis*, and *C. sinensis.* ^31^ ^,^ ^34^ ^,^ ^37^ ^,^ ^38^ On the other hand, this mouse strain is susceptible to *H. polygyrus* and *L. sigmodontis*.^32^ ^,^ ^35^ Infection with the fungus *Pneumocystis* led to a strong innate immune response and did not trigger the activation of a protective humoral response.^40^

Up to the present moment, no studies have used FVB/NJ mouse for *Leishmania* infections. Therefore, the aim of the current study was to characterise the FVB/NJ mouse as another model for cutaneous infection by *Leishmania (L.) amazonensis*, one of the main causative agents of TL in Brazil. For this purpose, *in vitro* experiments were conducted to assess parasite phagocytosis and infection kinetics in macrophages, and *in vivo* infections were performed to evaluate the development of footpad lesions and parasite burden. Our data demonstrate that the FVB/NJ strain displays infection patterns similar to BALB/c mice both *in vitro* and *in vivo*. These results, along with the docile behaviour and vigorous reproductive performance, with generation of large litters (between 9 and 13 animals), render the FVB/NJ mouse a promising and suitable infection model for tegumentary leishmaniasis.

## MATERIALS AND METHODS

*Ethical statement* - Experiments with FVB/NJ and BALB/c mice were performed according to the Brazilian College of Animal Experimentation (CONEP) guidelines and with the approval of the Institutional Animal Care and Use Committee (CEUA) of the University of São Paulo (protocol number 2314160519).

*Leishmania (L.) amazonensis culture* - Promastigotes of *L. (L) amazonensis* from the strain LV79 (MPRO/72/M1841) and M2269 (MHOM/BR/1973/M2269) expressing luciferase (*La*-LUC) were cultured at 24ºC in complete medium 199 (pH 7.2) (Gibco, Invitrogen) containing 40 mM HEPES (pH 7.4), 0.1 mM adenine, 0.0005% hemin, 20 μg/mL gentamicin, and 10% foetal bovine serum (FBS). For M2269 *La*-LUC the culture was supplemented with 32 µg/mL of geneticin (G418). The cultures were passaged weekly to a density of 2 x 10^6^ parasites/mL and used in the stationary phase.

*Bone marrow-derived macrophages (BMDM)* - BALB/c and FVB/NJ mice were euthanised in a CO_2_ chamber. Cells were collected from the bone marrow and differentiated as previously described.^42^ A total of 2 x 10^5^ or 4 x 10^5^ macrophages/well were plated in RPMI 1640 (pH 7.2) with 10% serum in 48- or 24-well plates on 9 mm or 13 mm glass coverslips, respectively, in a 37ºC incubator with 5% CO_2_ until the next day.

*Peritoneal macrophages* - Resident peritoneal macrophages were obtained from BALB/c and FVB/NJ animals as previously described.^20^ Cells were suspended in RPMI and transferred to 48- or 24-well plates with glass coverslips. After 2 h of incubation in a 37ºC incubator with 5% CO_2_, the medium was changed to RPMI supplemented with 10% FBS and 20 μg/mL gentamicin, and cells were incubated overnight at 37ºC with 5% CO_2_.

*Infection of murine macrophages* - Murine macrophages derived from BM precursors or recovered from the peritoneal cavity were plated and infected as previously described.^20^ At the end of the experiment, coverslips were fixed with methanol for 10 min, stained with the Instant Prov Kit (Newprov) and mounted on slides using Entellan (Merck). One hundred macrophages per coverslip (in triplicate) were analysed, and the percentage of infected macrophages and the mean number of amastigotes per infected macrophage were determined.

*Phagocytosis of promastigotes by murine macrophages* - Murine BMDM from BALB/c and FVB/NJ were plated on 13 mm coverslips in 100 mm culture dishes. Stationary-phase promastigotes were added at a multiplicity of infection (MOI) of 1:10 and plates were kept at 4ºC on ice for 2 h. Subsequently, plates were transferred to a 34ºC incubator with 5% CO_2_ for 5 min. Cells were thoroughly washed with sterile PBS and fixed with 2% paraformaldehyde (PFA) for 1 h. They were then quenched with 50 mM ammonium chloride for 10 min and washed with PBS for 10 min. Coverslips were blocked with 2% bovine serum albumin (BSA) in PBS for 30 min and incubated overnight with anti-*Leishmania* mouse serum at a dilution of 1:300 in 0.2% BSA-PBS. After washing with PBS, they were incubated for 1 h with anti-mouse IgG (H+L) 488 Alexa Fluor (1:200) and DAPI (1:1000) in sterile PBS. After washing, coverslips were mounted with ProLong (Molecular Probes) for analysis under fluorescence microscopy. Images were captured using an inverted fluorescence microscope model AxioVert.A1 (Carl Zeiss) and analysed using the ZEISS ZEN 3.7 software. A total of 100 macrophages were analysed in terms of numbers of adhered (green) and internalised parasites (blue, labelled with DAPI).

*Phagocytosis of zymosan by murine macrophages* - Zymosan particles (Molecular Probes) were suspended at 1 mg/mL (1x10^7^ particles/mL) in RPMI medium pH 7.2 supplemented with 10% FBS. The zymosan phagocytosis experiments were conducted similarly to those with *Leishmania*. Cells were incubated at a ratio of two zymosan particles per cell for 1 h, fixed with absolute methanol for 5 min, stained with Giemsa and mounted as described in infection assays.

*In vivo infection of FVB/NJ and BALB/c mice for analysis of lesion size and parasite burden through in vivo imaging* - The FVB/NJ strain was kindly provided by Prof Francisco Laurindo from INCOR, School of Medicine, University of São Paulo (FM-USP). Mice from FVB/NJ (10) and BALB/c (10) strains, aged four to eight weeks, were infected with 2 × 10^6^ promastigotes of *L. (L.) amazonensis* MHOM/BR/1973/M2269 expressing luciferase (*La*-LUC), as previously described by our group.^20^ The progression of lesions and imaging were monitored weekly for 12 weeks and analysed as described.^20^

The comparison between the expected and observed number of infected animals in each group was calculated using the Fisher’s Exact Test. Animals that did not show infection after 12 weeks were not submitted to quantification of parasite burden.

*Complement-mediated lysis assay* - Blood samples from BALB/c and FVB/NJ mice were collected and incubated at 4ºC for 15 min. Tubes were centrifuged at 1500 ×g at 4ºC for 10 min, the sera were collected and pooled, and stored at -80ºC. Sera from three different pools of each mouse strain were read at 595 nm and those with the optical densities (ODs) between 0.03 and 0.04 were chosen. Lysis and MTT (5 mg/mL) assays were conducted following the published protocol,^43^ except that parasite incubations with serum were carried out at 34ºC.

## RESULTS

**Fig. 1:**
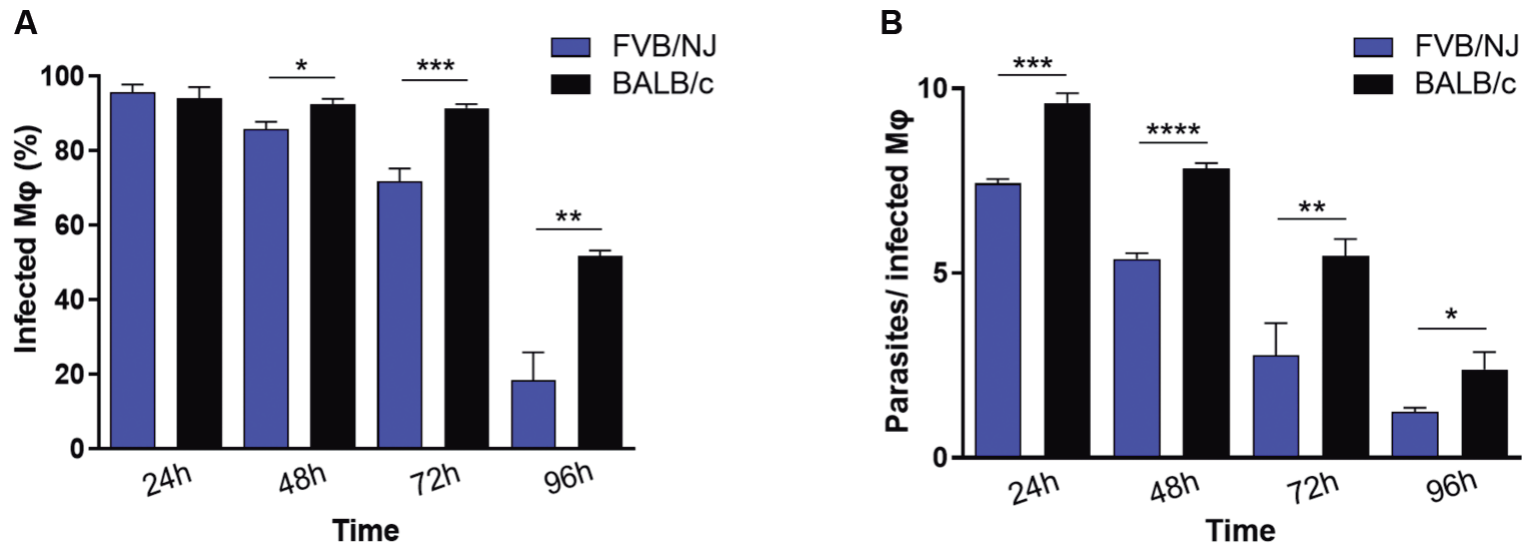
infection of bone marrow-derived macrophages (BMDM) from Friend Virus B NIH Jackson (FVB/NJ) and BALB/c with *Leishmania (Leishmania) amazonensis* promastigotes (MOI 1:10) for 24, 48, 72, and 96 h. The percentage of infected macrophages (A) and the number of parasites per infected macrophage (B) were determined. Results from a representative experiment out of three with similar profiles. Statistical analysis: Student’s t-test between FVB/NJ and BALB/c at each time point. *p ≤ 0.05. **p ≤ 0.01. ***p ≤ 0.001. ****p ≤ 0.0001.

*In vitro infection of BMDM and peritoneal macrophages from FVB/NJ and BALB/c with L. (L.) amazonensis* - BMDM and peritoneal macrophages from FVB/NJ and BALB/c were infected with *L. (L.) amazonensis* for 24, 48, 72, and 96 h to characterise the infection over time in FVB/NJ macrophages. BMDM of BALB/c showed a higher percentage of infection starting from 48 h (Fig. 1A) compared with those from FVB/NJ. Regarding parasites per macrophage, those from BALB/c exhibited significantly higher numbers of amastigotes per cell at all time points (24 to 96 h) (Fig. 1B), indicating that FVB/NJ macrophages might either phagocytise fewer promastigotes or control intracellular parasites, preventing their survival or differentiation into amastigotes. In peritoneal macrophages, there was no statistically significant difference between the strains in terms of infection percentage, although BALB/c macrophages were slightly more infected than FVB/NJ macrophages [Supplementary data (Fig. 1A)]. In terms of parasites per macrophage, a significant difference was only observed at the 48-h time point, when BALB/c peritoneal macrophages exhibited a higher number of parasites per cell. In other time points, BALB/c peritoneal macrophages had slightly more intracellular parasites [Supplementary data (Fig. 1B)].

**Fig. 2:**
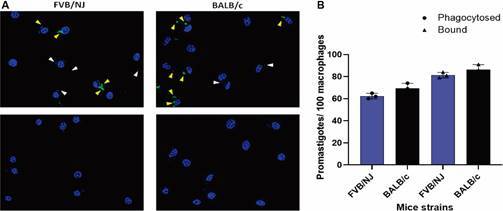
phagocytosis of *Leishmania (Leishmania) amazonensis* promastigotes by bone marrow-derived macrophages (BMDM) from Friend Virus B NIH Jackson (FVB/NJ) and BALB/c. (A) Representative images of the phagocytosis experiment. White arrows indicate internalised promastigotes, labelled with DAPI (blue), and yellow arrows indicate adhered promastigotes, labelled with Alexa 488 (green) (upper images). Parasite-unlabelled images represent the control without anti-*Leish* serum (lower images). Macrophage nuclei are stained with DAPI. Images were processed by deconvolution. (B) Number of bound and phagocytosed promastigotes per 100 macrophages after 5 min. Results from a representative experiment out of three with similar profiles. Student’s t-test between bound to FVB/NJ and BALB/c and between phagocytosed by FVB/NJ and BALB/c.

*Phagocytosis of promastigotes by FVB/NJ and BALB/c macrophages* - We demonstrated that BALB/c BMDMs had significantly higher numbers of amastigotes per cell compared with FVB/NJ at 24-, 48-, 72-, and 96-h post-infection. This suggests that BALB/c macrophages either phagocytise more promastigotes or that FVB/NJ macrophages kill more parasites after phagocytosis. To address this question, we compared the adhesion and phagocytosis of promastigotes by BMDM from FVB/NJ and BALB/c strains. As shown in Fig. 2, BALB/c macrophages exhibited statistically similar number of internalised promastigotes compared with FVB/NJ, indicating that differences in phagocytosis do not explain the higher infection observed in BALB/c macrophages.

**Fig. 3:**
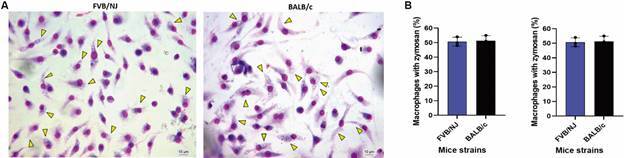
phagocytosis of zymosan particles (yellow arrows) by bone marrow-derived macrophages (BMDM) from Friend Virus B NIH Jackson (FVB/NJ) and BALB/c (A). Percentage of macrophages with zymosan (left) and number of particles phagocytosed by macrophage (right) after 1 h (B). Results from a representative experiment out of three with similar profiles. Student’s t-test.

*Phagocytosis of zymosan by FVB/NJ and BALB/c macrophages* - With the aim of evaluating the process of phagocytosis by FVB/NJ and BALB/c macrophages, cells were incubated with zymosan particles for 1 h. Fig. 3 shows that BALB/c macrophages phagocytic capacity (Fig. 3A-C).

**Fig. 4:**
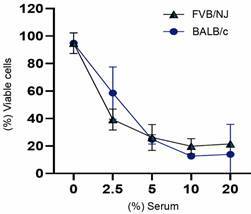
resistance of *Leishmania (Leishmania) amazonensis* promastigotes (Day 4) to Friend Virus B NIH Jackson (FVB/NJ) and BALB/c complements. The percentage of viable cells relative to the control (without serum) after 30 min incubation at 34**º**C in the presence of 2.5%, 5%, 10%, and 20% serum pooled from mice of the two strains. Assays performed in technical triplicates. Statistical analysis: Student’s t-test. Results are from a representative experiment out of three with similar profiles.

*Resistance of promastigotes to FVB/NJ and BALB/c complement* - As already mentioned, FVB/NJ strain has a deficiency in the complement factor C5 and in the expression of C3 convertase. This prompted us to compare the resistance of *L. (L.) amazonensis* promastigotes to FVB/NJ and BALB/c sera. Strikingly, the results shown in Fig. 4 indicate that sera from the two mouse strains have similar lytic capacity, despite the known complement deficiency of FVB/NJ mice.

*In vivo infection of FVB/NJ and BALB/c strains with L. (L.) amazonensis* - To characterise cutaneous lesion development and parasite burden over 12 weeks, FVB/NJ and BALB/c mice were infected in the footpad with *L. (L.) amazonensis* expressing luciferase (*La*-LUC). Lesion size was measured weekly, and parasite burden was estimated weekly by luminescence.

**Fig. 5:**
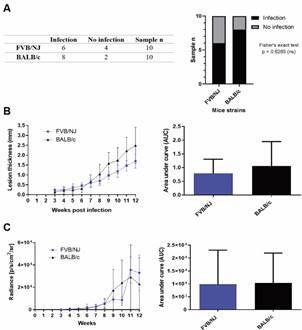
lesion development and parasite load of Friend Virus B NIH Jackson (FVB/NJ) and BALB/c mice (10 animals in each group) infected with *Leishmania (Leishmania) amazonensis* MHOM/BR/1973/M2269 *La*-LUC. (A) Number of animals with (infection) and without (no infection) active infection 12 weeks after inoculation. Statistical analysis by Fisher’s exact test. (B) Footpad lesion thickness of FVB/NJ and BALB/c mice over 12 weeks. Lesion development (left) and area under curve (AUC) (right). Statistical analysis: Student’s t-test for AUC. (C) Parasite burden calculated by radiance from the region of interest (ROI) (luminescence) in FVB/NJ and BALB/c mice over 12 weeks. The difference between photon emissions (lesion - uninfected footpad) was calculated and expressed in photons/s/cm2/sr. The same ROI was applied to all animals. Radiance (left) and AUC (right). Statistical analysis: Student’s t-test for AUC.

The number of animals that developed or not active infection 12 weeks after inoculation was analysed, and only luminescence from animals with active infection was quantified. As shown in Fig. 5A, six animals from FVB/NJ and eight from BALB/c showed active infection. Fisher’s exact test indicated no statistical difference between the two strains.

As shown in Fig. 5B-C, there was also no statistical difference in lesion size and in parasite burden between the two strains over the course of 12 weeks, although BALB/c developed slightly larger lesions after the seventh week of infection.

## DISCUSSION

No study up to now employed FVB mice in *Leishmania* infections. SWR/J mice, from which FVB/NJ was derived, have rarely been used as model for *Leishmania* infection.^44^ *In vitro* infection of BMDM and peritoneal macrophages with *L. (L.) infantum* (*syn L. (L.) chagasi*) showed that BALB/c macrophages are more susceptible to infection and more permissive to amastigote replication than SWR/J macrophages, with no difference in cytokine production.^45^ Our observations that FVB/NJ BMDM are less infected by *L. (L.) amazonensis* than BALB/c seem similar to the results obtained for SWR/J macrophages.

Our *in vivo* infections indicated that FVB/NJ is permissive to infection by *L. (L.) amazonensis*. *In vivo* infection in the footpad of BALB/c and SWR/J with the Isabel-NIH strain of *L. (L.) amazonensis*, diffuse leishmaniasis, resulted in lesions that became necrotic in both strains, and the loss of the affected footpad was frequently observed after 14 weeks.^46^ In the present study, lesions did not lead to footpad loss neither in FVB/NJ nor in BALB/c strains after 12 weeks. Indeed, we have previously shown that infection with *L. (L.) amazonensis* LV79 strain for 13 weeks did not lead to footpad loss.^20^ ^,^ ^47^

SWR/J mice were more effective in controlling *in vivo* infection by *L. (L.) major* compared to BALB/c mice and to animals resulting from a cross between the two strains.^44^ Infection of SWR/J and C57BL/6 mice with *L. (L.) major* exhibited a similar pattern of lesion development, which regressed after the fourth week, and these two strains have a similar cytokine production pattern, with high levels of IFN-γ and low levels of IL-4.^48^ Another study reported that *in vivo* infection with *L. (L.) major* may show differences between BALB/c and SWR/J mice depending on the site of inoculation. SWR/J mice exhibited higher ability to control infections in the footpad than BALB/c mice, and ulcerated lesions were observed after the eighth week of infection in BALB/c footpads. From the eleventh week on lesions in SWR/J mice started to resolve, with the presence of small, ulcerated lesions, while BALB/c lesions did not regress. BALB/c mice had significantly higher numbers of parasites in the footpad, spleen, and draining lymph nodes compared with SWR/J mice. The parasite burden followed the disease progression profile for both mice strains.^49^

In the present study, BALB/c mice also developed slightly larger lesions in the footpad compared with FVB/NJ mice from the seventh week of infection on, although with no statistical differences. The parasite burden followed the disease progression profile (lesion thickness) until the tenth week. Curiously, the known C5-deficiency of FVB/NJ mice had no impact in parasite survival in *in vivo* infections. Accordingly, and different from our expectations, we observed no differences in promastigote survival after incubations with BALB/c and FVB/NJ serum.

This work shows that FVB/NJ mouse strain is promising as a model for studying tegumentary leishmaniasis by *Leishmania (L.) amazonensis*. Indeed, its similarity with the BALB/c strain (the most frequently used murine model for *Leishmania* infection) in terms of phagocytosis and *in vivo* infections suggests that it might be another murine strain susceptible to this *Leishmania* species, useful for analysing different aspects of cutaneous localised leishmaniasis. The use of this novel model also paves the way for further studies aimed at understanding the pathogenesis of lesions caused by *L. (L.) amazonensis* and other dermotropic species of *Leishmania*. This is especially pertinent given the widespread use of FVB/NJ as a transgenic model in several disease pathogenesis studies.
