# Supplementary material for: FVB/NJ strain as a mouse model for cutaneous leishmaniasis by Leishmania (L.) amazonensis
Source: Mem Inst Oswaldo Cruz. 2024 Mar 15;119:e230182. doi: 10.1590/0074-02760230182 (PMC10941652; doi:10.1590/0074-02760230182)
Supplement: Supplementary file 1 [file 1678-8060-mioc-119-e230182-s.pdf]

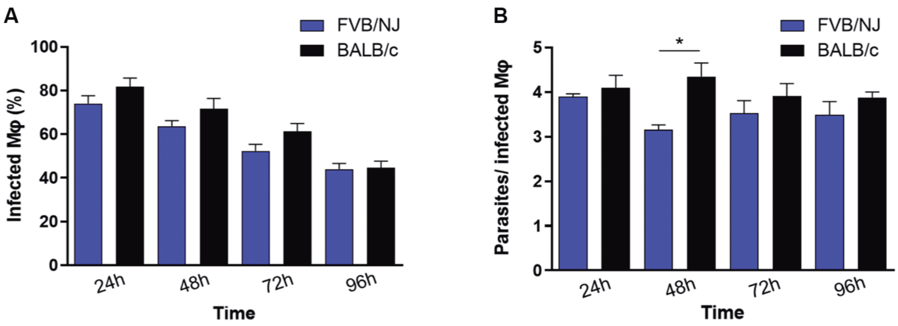

Infection assays of peritoneal macrophages from Friend Virus B NIH Jackson (FVB/NJ) and BALB/c with *Leishmania (Leishmania) amazonensis* (MOI 1:10) for 24, 48, 72, and 96 h. The percentage of infected macrophages (A) and the number of parasites per infected macrophage (B) were determined. Results are from a representative experiment out of three with similar profiles. Statistical analysis: Student's t-test between FVB/NJ and BALB/c at each time point. *$p \leq 0.05$.

CC BY
